# c-MycER^TAM ^transgene silencing in a genetically modified human neural stem cell line implanted into MCAo rodent brain

**DOI:** 10.1186/1471-2202-10-86

**Published:** 2009-07-21

**Authors:** Lara Stevanato, Randolph L Corteling, Paul Stroemer, Andrew Hope, Julie Heward, Erik A Miljan, John D Sinden

**Affiliations:** 1 ReNeuron Limited, Surrey Research Park, 10 Nugent Road, Guildford, Surrey, GU2 7AF, UK

## Abstract

**Background:**

The human neural stem cell line CTX0E03 was developed for the cell based treatment of chronic stroke disability. Derived from fetal cortical brain tissue, CTX0E03 is a clonal cell line that contains a single copy of the c-mycER^TAM ^transgene delivered by retroviral infection. Under the conditional regulation by 4-hydroxytamoxifen (4-OHT), c-mycER^TAM ^enabled large-scale stable banking of the CTX0E03 cells. In this study, we investigated the fate of this transgene following growth arrest (EGF, bFGF and 4-OHT withdrawal) *in vitro *and following intracerebral implantation into a mid-cerebral artery occluded (MCAo) rat brain. *In vitro*, 4-weeks after removing growth factors and 4-OHT from the culture medium, c-mycER^TAM ^transgene transcription is reduced by ~75%. Furthermore, immunocytochemistry and western blotting demonstrated a concurrent decrease in the c-MycER^TAM ^protein. To examine the transcription of the transgene *in vivo*, CTX0E03 cells (450,000) were implanted 4-weeks post MCAo lesion and analysed for human cell survival and c-mycER^TAM ^transcription by qPCR and qRT-PCR, respectively.

**Results:**

The results show that CTX0E03 cells were present in all grafted animal brains ranging from 6.3% to 39.8% of the total cells injected. Prior to implantation, the CTX0E03 cell suspension contained 215.7 (SEM = 13.2) copies of the c-mycER^TAM ^transcript per cell. After implantation the c-mycER^TAM ^transcript copy number per CTX0E03 cell had reduced to 6.9 (SEM = 3.4) at 1-week and 7.7 (SEM = 2.5) at 4-weeks. Bisulfite genomic DNA sequencing of the *in vivo *samples confirmed c-mycER^TAM ^silencing occurred through methylation of the transgene promoter sequence.

**Conclusion:**

In conclusion the results confirm that CTX0E03 cells downregulated c-mycER^TAM ^transgene expression both *in vitro *following EGF, bFGF and 4-OHT withdrawal and *in vivo *following implantation in MCAo rat brain. The silencing of the c-mycER^TAM ^transgene *in vivo *provides an additional safety feature of CTX0E03 cells for potential clinical application.

## Background

Stem cell therapy is a facet of regenerative medicine that aims to ameliorate the damage caused to the brain by the grafting of healthy "reparative" cells. Pioneering studies implanting mouse neural stem cells (NSCs) into the brains of stroke animals have demonstrated significant recovery in motor and cognitive tests [1-5]. These findings provide a rational approach to the development of a cell based therapy for ischemic stroke. A substantial and consistent supply of allogeneic NSCs is required in order to treat a large patient population. Unfortunately, human NSCs are somatic stem cells and susceptible to genetic and phenotypic changes and loss of biological activity following extensive tissue culture expansion [6-8].

Immortalization of NSCs overcomes their restricted expansion potential. The myc proto-oncogene has proven a successful tool for immortalization of NSCs [9-16]. In these research applications, both v- and c-myc promote consistent and enhanced tissue culture expansion of human NSCs while maintaining a stable karyotype and retaining biological activity. The c-mycER^TAM ^technology has proven to be as successful in generating immortalized neural stem cell lines as c-myc alone [17-20]. The c-mycER^TAM ^transgene is better suited as an immortalizing agent for clinical applications because c-Myc protein function is conditional on the presence of the tamoxifen metabolite, 4-hydroxytamoxifen (4-OHT). The translated c-MycER^TAM ^recombinant protein is a conjugation of human c-Myc and modified mouse estradiol receptor (ER) and is present as an inactive monomer in the cytosol of the cell. When added to the tissue culture medium, 4-OHT specifically binds to the ER moiety causing the c-MycER^TAM ^protein to dimerize and subsequently translocate to the cell nucleus where c-Myc is active as a transcription factor [21].

CTX0E03 is a human NSC line that was derived using c-mycER^TAM ^technology and showed recovery in sensorimotor deficits following grafting in MCAo rodent brain [19,22]. One copy of the c-mycER^TAM ^transgene is integrated into the CTX0E03 cell genome (chromosome 13) under the cytomegalovirus (CMV) immediate early promoter [19]. We have shown that the c-mycER^TAM ^transgene is expressed in proliferating CTX0E03 cell cultures *in vitro*; however, the expression of this transgene following the grafting of CTX0E03 cells *in vivo *was not characterized. Although neoplasm formation has never been observed with CTX0E03 cells in multiple pre-clinical studies, this information would nonetheless be important in assessing the inherent risk of using a genetically modified cell line for clinical applications.

Analysis of transgene expression following implantation of genetically modified cells is challenging because of the relatively small proportion of implanted cells compared to the host. Using a fluorescent reporter transgene, the CMV promoter has been shown to undergo silencing in grafted hematopoietic cells *in vivo *[23]. In another example, Lee *et al*. demonstrated by immunohistochemistry (IHC) that v-myc was silenced following intracerebral implantation of immortalized HBF1.3 cells [24]. Unfortunately, these methodologies were not appropriate for our application. The c-mycER^TAM ^transgene in CTX0E03 cells does not contain a fluorescent reporter that could be monitored. With respect to IHC, we were concerned it would be inconclusive due to the unknown sensitivity and potential cross-reactivity of the antibodies. Furthermore, IHC was ruled out since the analysis would be limited to a finite number of sections and would not encompass all cells present in the graft.

In this study, we developed a novel and sensitive method that is able to analyze absolute gene expression within human genetically modified cells grafted into rodent brain. This method used the human specific Alu genomic sequence to detect and quantify CTX0E03 cells grafted within the rodent brain background [25,26]. Absolute quantification of c-mycER^TAM ^transcription made it possible to calculate the number of c-mycER^TAM ^transcript copies per CTX0E03 cell. Using this methodology, c-mycER^TAM ^transgene silencing was shown following grafting of CTX0E03 cells into MCAo rat brain. Although not intentionally incorporated into the design technology of c-mycER^TAM^, silencing of the CMV transgene promoter *in vivo *demonstrates an additional safety feature of this technology and of the CTX0E03 cells.

## Results

### Alu and c-mycER^TAM ^assay design

**Figure 1:**
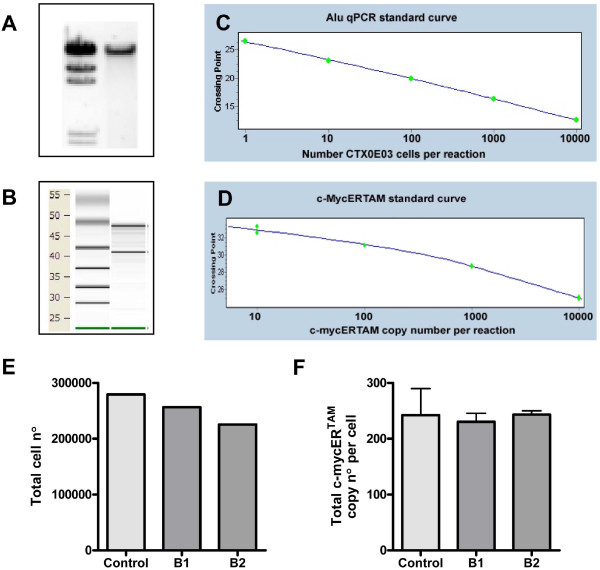
**Alu sequence and c-mycER^TAM ^assay development and validation**. Gels showing the quality and purity of gDNA by agarose gel electrophoresis (A) and RNA by virtual gel produced by Agilent 2100 Bioanalyzer (B, RNA Integrity Number >9.4 as analyzed by Agilent RNA 600 nano kit [32]) isolated from the same sample. Standard curves used to determine: cell number by Alu sequence qPCR (C, Error 0.0300, efficiency 1.993; 3 replicates); absolute *c*-mycER^TAM ^copy number by *c*-mycER^TAM ^qRT-PCR (D, Error 0.0837 and efficiency 2.131; 3 replicates). All standard curves were generated from CTX0E03 gDNA diluted in rat gDNA or cDNA. Crossing point refers to the number of PCR cycles required to generate a detectable fluorescent signal generated on a Roche LC480 instrument. Positive control rat brain samples (B1 and B2) were grafted with approximately 300,000 CTX0E03 cells each and harvested immediately (E, F). Data shown is the total number of CTX0E03 cells in each tissue section as determined by Alu, where control is the number of viable cells in the cell suspension prior to injection as determined by counting using a haemocytometer (E); and total c-mycER^TAM ^transcript copy number calculated per CTX0E03 cell detected in brain samples, where control is the number of copies per cell detected *in vitro *culture (F).

The analysis of the *c*-mycER^TAM ^transcript expression per CTX0E03 cell, both *in vivo *and *in vitro*, was dependent upon the ability to sequentially purify genomic DNA (gDNA) and RNA from the same biological sample using the All DNA/RNA Kit (Qiagen). The gDNA preparation yielded one single band by agarose gel electrophoresis with no evidence of degradation or contaminating RNA (Figure 1A). The RNA preparation yielded the expected 28S and 18S bands, again with no degradation or DNA contamination visible (Figure 1B). Standard curves were then generated to determine: 1) the number of CTX0E03 cells present in a gDNA sample by Alu qPCR (Figure 1C); and 2) the number of c-mycER^TAM ^transcripts present in a retro-transcribed RNA reaction sample (Figure 1D). The Alu sequence assay could reproducibly detect human CTX0E03 gDNA with as few as one CTX0E03 cell per reaction; whereas, the c-mycER^TAM ^assay could detect as few as 10 copies per reaction.

### Validation of Alu and c-mycER^TAM ^assays

The Alu sequence and c-mycER^TAM ^transcription assays were developed in the presence of rat brain gDNA or cDNA, as described in the *Methods*. These experiments involved mixing purified CTX0E03 DNA/cDNA with purified rat brain DNA/cDNA. However, in order to verify that CTX0E03 cells grafted in rat brain could be processed and analysed effectively together a positive control experiment was carried out. This experiment consisted of harvesting and processing the rat brain tissue immediately after a 6 μl injection of CTX0E03 cells was delivered. It was determined by counting using a haemocytometer that the cell suspension prior to delivery contained 46,600 cells/μl – i.e. total of 279,600 cells were present in 6 μl loaded into the syringe. By Alu sequence analysis, the injected rat brain tissue sections contained an average of 241,116 (SEM = 15,487) CTX0E03 cells (Figure 1F). The measured number of CTX0E03 cells is within the range expected. Analysis of the total RNA isolated from these samples showed CTX0E03 cells to express on average 236.9 (SEM = 7.8) c-mycER^TAM ^transcript copies per cell (Figure 1G). Control CTX0E03 cells measured in culture were shown to have a similar value of 242.3 (SEM = 47.8) c-mycER^TAM ^transcript copies per cell.

### CTX0E03 cell detection and quantification *in vivo*

**Figure 2:**
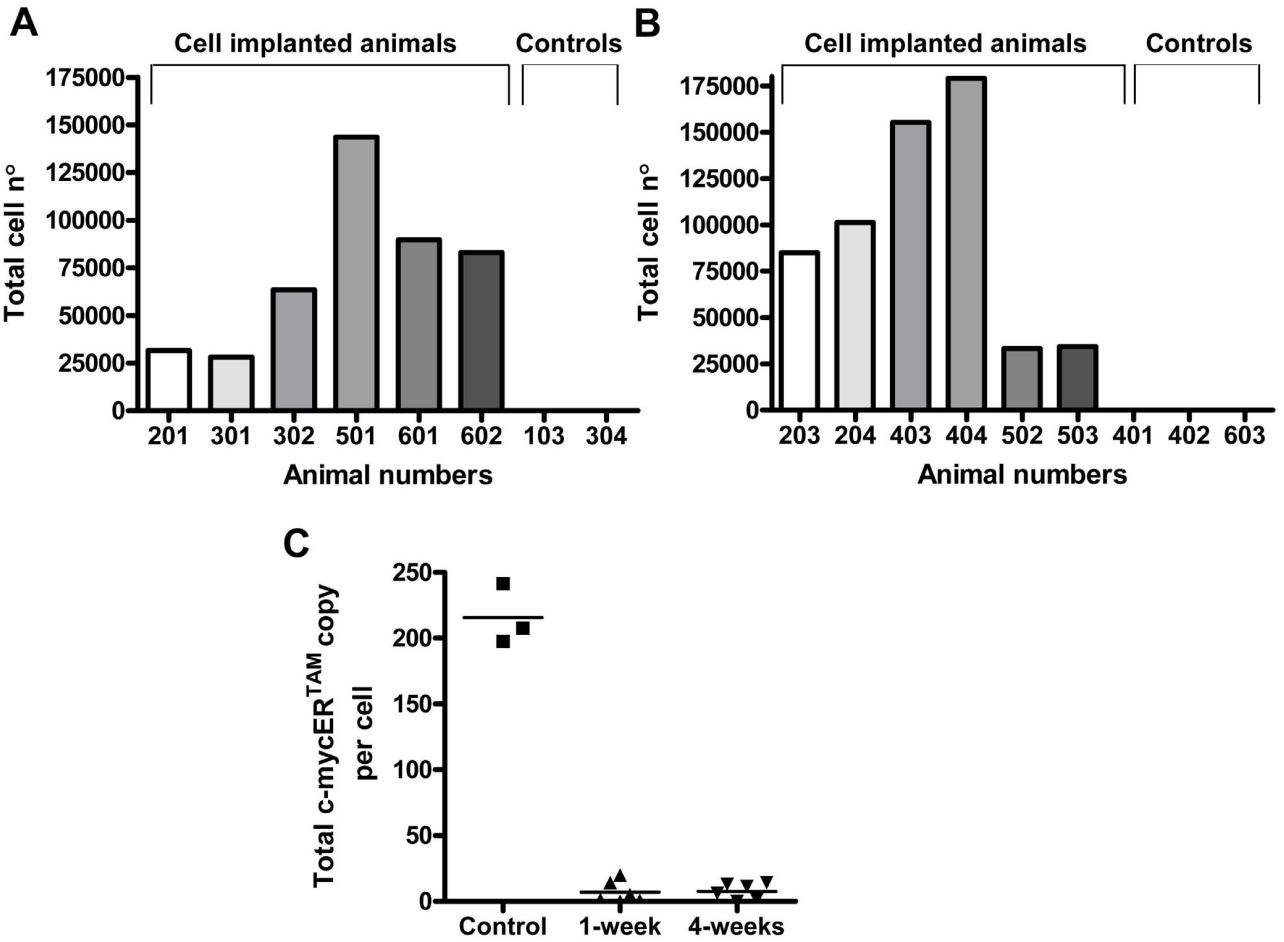
**Detection of CTX0E03 cells and c-mycER^TAM ^transcription in grafted rat brain**. Total cells found at 1-week (A) and 4-weeks (B) post-implantation by Alu qPCR. Animal numbers 103, 304, 401, 402 and 603 were vehicle injected only control brain samples. Absolute quantification of c-mycER^TAM ^transcript level per CTX0E03 cell, in cell suspensions prior to implantation (control) and *in vivo *(C).

Four weeks after the MCAo, a total of 450,000 CTX0E03 cells were implanted bilaterally into the putamen. The rats were sacrificed 1-week or 4-weeks post-implantation, their brains were removed and sections encompassing the injection tracts were dissected and snap frozen in liquid nitrogen. The Alu and c-mycER^TAM ^assays were then carried out on the gDNA and cDNA isolated from each tissue section, respectively. CTX0E03 cells were detected in all implanted animals at both time points, with a range of 28,151 to 179,184 CTX0E03 cells detected per brain. The total number of CTX0E03 cells detected, per rat brain, is shown at 1-week (Figure 2A) and 4-weeks (Figure 2B) post-implantation. An aliquot of the CTX0E03 cell suspension prepared for implantation was retained and used to determine the pre-implantation c-mycER^TAM ^transcript copy number. The average c-mycER^TAM ^transcription level for the three CTX0E03 cell suspensions prepared in this study was found to be 215.7 (SEM = 13.2) copies per cell (Figure 2C). This is compared to the population of CTX0E03 cells detected *in vivo*, where the average number of c-mycER^TAM ^transcript copies per CTX0E03 cell was calculated to be 6.9 (SEM = 3.4) at 1-week and 7.7 (SEM = 2.5) at 4-weeks (Figure 2C). The number of CTX0E03 cells, the number c-mycER^TAM ^transcripts calculated per tissue section and the resultant number of c-mycER^TAM ^transcript copies per CTX0E03 cell, are shown in Table 1. c-MycER^TAM ^transcripts were detected within approximately 50% of the tissue sections containing CTX0E03 cells at both time points. It is important to note, in no instance was a c-mycER^TAM ^transcript signal detected where CTX0E03 cells were absent.

**Table 1:** c-MycER^TAM ^transcript copy number and number of CTX0E03 cells calculated in the grafted rat brain sections at 1- and 4-weeks post-implantation

**Week 1**					**Week 4**				
**Rat# region**	**Total c-** **mycER^TAM^** **copy #**	**Total cell #** **Alu assay**	**Total *c*** **-mycER^TAM ^** **copy #/Total cell** **# Alu assay**	***c*-mycER^TAM^** **copy # per cell** **per animal**	**Rat# region**	**Total *c*** **-mycER^TAM^** **copy #**	**Total cell** **# Alu assay**	**Total *c*** **-mycER^TAM ^** **copy #/Total cell** **# Alu assay**	***c*-mycER^TAM ^** **copy # per cell** **per animal**

**201 F1**	N.D.^1^	N.D.^2^	N/A		**203 F1**	N.D.	N.D.	N/A	

**201 F2**	N.D.	1066.9	0.0		**203 F2**	N.D.	N.D.	N/A	

**201 FN1**	18671.8	18821.5	1.0		**203 FN1**	N.D.	1776.9	0.0	

**201 FN2**	N.D.	11695.4	0.0	0.8	**203 FN2**	N.D.	15046.2	0.0	

**301 F1**	N.D.	530.8	0.0		**203 C**	521495.6	68123.1	7.7	6.1

**301 F2**	N.D.	356.8	0.0		**204 F1**	902695.4	45261.5	20.0	

**301 FN1**	N.D.	16269.2	0.0		**204 F2**	N.D.	5563.1	0.0	

**301 FN2**	N.D.	10993.9	0.0	0.0	**204 FN1**	N.D.	4850.8	0.0	

**302 F1**	N.D.	1075.4	0.0		**204 FN2**	418067.7	45661.5	9.2	13.0

**302 F2**	N.D.	837.7	0.0		**403 F1**	N.D.	N.D.	N/A	

**302 FN1**	909865.9	42992.3	21.1		**403 F2**	264100.0	5501.5	48.0	

**302 FN2**	N.D.	18664.6	0.0	14.3	**403 FN1**	388332.3	85000.0	4.6	

**501 F1**	N.D.	N.D.	N/A		**403 FN2**	224224.0	17169.2	13.1	

**501 F2**	550.1	17846.2	0.0		**403 C**	1324893.5	47584.6	27.8	14.2

**501 C1**	2858994. 9	123000.0	23.2		**404 F1**	N.D.	2738.5	0.0	

**501 C2**	N.D.	2701.5	0.0	19.9	**404 F2**	31223.6	11076.9	2.8	

**601 F1**	N.D.	N.D.	N/A		**404 FN1**	N.D.	146750.0	0.0	

**601 F2**	N.D.	964.2	0.0		**404 FN2**	218605.5	15061.5	14.5	

**601 FN1**	10151.6	70061.5	0.9		**404 F ANT**	N.D.	3556.9	0.0	0.4

**601 FN2**	109810.0	18692.3	5.9	1.3	**502 F1**	N.D.	241.4	0.0	

**602 F1**	380824.6	41123.1	9.3		**502 F2**	N.D.	1046.2	0.0	

**602 F2**	N.D.	24076.9	0.0		**502 FN1**	150198.4	12776.9	11.7	

**602 C**	40085.1	11253.9	3.6		**502 FN2**	224974.8	19261.5	11.7	11.3

**602 FL**	N.D.	6541.5	0.0	5.1	**503 F1**	N.D.	N.D.	N/A	

					**503 F2**	N.D.	N.D.	N/A	

					**503 FN1**	N.D.	1230.8	0.0	

					**503 FN2**	N.D.	13292.3	0.0	

					**503 C**	N.D.	19692.3	0.0	

			**Mean**	**6.9**				**Mean**	**7.7**

			**SEM**	**3.4**				**SEM**	**2.5**

^1^N.D. Not Detected, signal was below the level of 10 c-mycER^TAM ^copies per reaction

^2^N.D. Not Detected, signal was below the level of one CTX0E03 cell per reaction

### Transgene methylation analysis

**Figure 3:**
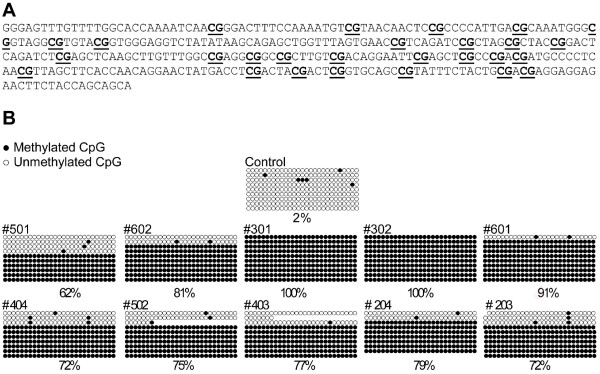
***In vivo *analysis of CpG methylation within the c-mycER^TAM ^promoter region in CTX0E03 cells**. CpG dinucleotide-containing regions (underlined) examined in CMV promoter of the c-mycER^TAM ^transgene (A). Methylation sequence analysis of the CMV transgene promoter region of *in vivo *and *in vitro *samples (B). Ten animal grafted brains were analyzed, five at 1-week (top set) and five at 4-weeks (bottom set), in addition, a non-implanted CTX0E03 cell sample (control). Ten clones containing the sequence depicted in (A) from each grafted rat brain were analyzed. Each row shown represents a clone. Filled circles = methylated CpG island; open circles = unmethylated CpG island. Percentages of global methylation are reported at the bottom of each sample.

Direct modification of the DNA by methylation is an epigenetic mechanism to downregulate or silence gene expression. Using bisulfite sequencing, the methylation status of the CMV transgene promoter was investigated in CTX0E03 cells pre- and post-implantation. The CMV promoter of the transgene contains numerous potential methylation sites within a CpG rich island (Figure 3A). Bisulfite sequencing of gDNA clones taken from control non-implanted CTX0E03 cells showed only 2% of these CpG sites were methylated (Figure 3B). However, the same analysis carried out on clones obtained from the CTX0E03 cell implanted MCAo rat brain samples showed that 86.8% (SEM = 7.1) and 75.0% (SEM = 1.4) of the CpG islands were methylated at 1-week and 4-weeks, respectively (Figure 3B). There was no statistical difference between 1- and 4-weeks, p = 0.14, indicating that the methylation had occurred within the first week and was constant to the 4-week time point. Collectively, only four gDNA transgene promoter clones *in vivo *were found to be devoid of any methylated sites from a total of 99 analysed; in other words, ~96% of the transgene promoter sequences analysed *in vivo *where found to contain at least one methylated CpG island.

### *In vitro *characterization

**Figure 4:**
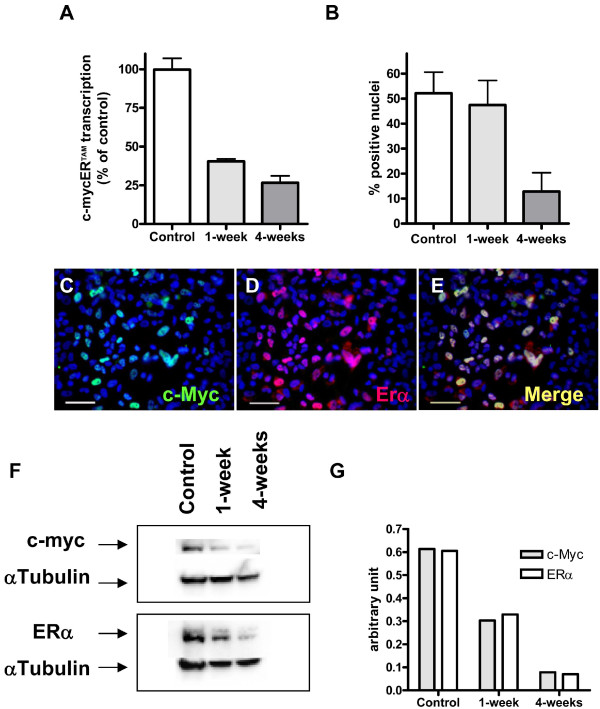
***In vitro *characterisation of c-mycER^TAM ^transcript and protein expression**. CTX0E03 cells were cultured in growth medium (control) and in non-growth promoting medium (in the absence of growth factors and 4-OHT) for 1- and 4-weeks. Evaluation of c-mycER^TAM ^gene transcript and protein levels were performed by qRT-PCR (A), ICC (B to E) and western blot (F). CTX0E03 cell ICC images shown in panels C to E are representative images of the control c-Myc (green), ERα (red) and overlay (Merge). Scale bars represent 50 μm. The western blots in panel F were quantified using densitometry normalised by α-tubulin (G).

Additional experiments were carried out *in vitro *to determine if a reduction in transgene transcription leads to a concomitant reduction in translated protein. Analyses were carried out on control CTX0E03 cells and those cultured for 1- and 4-weeks in the absence of growth factors and 4-OHT. In this non growth permissive condition, transgene transcription was found to decrease by 59.6% at 1-week and 73.4% at 4-weeks relative to control by qRT-PCR (Figure 4A). Analysis of c-MycER^TAM ^protein was performed by colocalization of c-Myc and ERα antigen staining by immunocytochemistry (ICC; Figure 4B, C, D, E). The c-Myc and ERα antigens were always found to colocalize in the nucleus of a proportion of CTX0E03 cells, indicating that the antibodies were detecting the c-MycER^TAM ^target (Figure 4E). Immunoreactive cells were counted and expressed as a proportion of total cells. These data showed control CTX0E03 cell cultures were approximately 52.0% positive for c-MycER^TAM^; whereas, following EGF, bFGF and 4-OHT withdrawal the number of CTX0E03 cells positive for c-MycER^TAM ^were 42.5% at 1-week and 12.8% at 4-weeks (Figure 4B). Western blot analysis using the c-Myc and ERα antibodies demonstrated that total c-MycER^TAM ^protein levels followed more closely the progressive drop observed by gene expression and were reduced by 53.8% at 1-week and 73.0% at 4-weeks following EGF, bFGF, and 4-OHT withdrawal (Figure 4F, G).

## Discussion

The c-mycER^TAM ^immortalization technology has been successfully utilized to derive a clonal human neural stem cell line from fetal cortical brain. This cell line, referred to as CTX0E03, possess biological attributes which are desirable for clinical applications for stroke therapy. Namely, this cell line can be continuously expanded *in vitro*, and following implantation in MCAo rodent brain, results in sensorimotor recovery in the absence of cell treatment-related histopathology [19,22]. The *in vitro *effects of c-mycER^TAM ^in the CTX0E03 cell line have been well characterized. Here we investigated the expression of this transgene following implantation of CTX0E03 cells into MCAo rat brain.

In this paper we report a novel method to examine absolute gene expression within human cells implanted into rodent brain. In this example, we analyzed the number of c-mycER^TAM ^transcript copies per CTX0E03 cell both *in vitro *and *in vivo*. The calculated c-mycER^TAM ^copies per CTX0E03 cell prepared directly from fresh culture (242.3; SEM = 47.8), cell suspension prior to implantation (215.7; SEM = 13.2) or immediately following implantation (236.9; SEM = 7.8) had comparable values. This validated that the assays employed were CTX0E03 cell specific and the rodent brain background did not cause any interference. Using these established assays, c-mycER^TAM ^transgene transcription was analyzed in the surviving CTX0E03 cell population at 1- and 4-weeks post-implantation in MCAo rodent brain. At both time points there was a substantial reduction in the calculated number of c-mycER^TAM ^transcript copies per CTX0E03 cell detected.

This molecular biological approach provided many advantages over immunohistochemistry and *in situ *hybridization techniques. The results obtained by RT-PCR and PCR were quantitative. Immunohistochemistry or *in situ *hybridization techniques do not permit the quantitative assessment of c-mycER^TAM ^levels within a given cell. In addition, the sensitivity of molecular biological techniques were defined, as a single CTX0E03 cell and ten copies of c-mycER^TAM ^per reaction, respectively; whereas, this level of sensitivity would have been difficult, if not impossible, to achieve by immunohistochemistry or *in situ *hybridization techniques. Our *in vivo *samples were processed rapidly, immediately after harvesting, thus capturing the most accurate data. Furthermore, from a practical perspective this molecular biological approach analyzed all CTX0E03 cells present in the rodent brain, whereas, histological preparation methods would require a large number of sections to analyze all cells present. Although the average transgene expression significantly decreased *in vivo*, we cannot exclude the possibility that the expression observed may have arisen from a limited number of CTX0E03 cells. However, the silencing of c-mycER^TAM ^expression observed *in vivo *is consistent with the absence of tumor formation derived from CTX0E03 cells when implanted *in vivo *[19,22].

Transgene silencing can occur at a transcriptional or post-transcriptional level. Long-term transcriptional silencing of retrovirus-delivered genes has been shown largely to result from methylation of the cytosine nucleotides within the promoter regions [27]. The factors responsible for DNA methylation is reviewed in detail elsewhere [28]. As the c-mycER^TAM ^transgene was delivered into the CTX0E03 cell line using a retrovirus, it was reasonable to speculate that transgene silencing had occurred through methylation of CpG islands within the transgene promoter region. Bisulfite DNA sequencing was used to determine the methylation status of the CMV promoter region of the c-mycER^TAM ^transgene within CTX0E03 cells. In culture, only 6 of the total CpG sites screened were found to be methylated. In the samples prepared from CTX0E03 grafted MCAo rodent brain 62 to 100% of the sites were methylated. These data unequivocally show that c-mycER^TAM ^expression is silenced at a transcriptional level *in vivo *as a result of DNA methylation within the CMV transgene promoter region.

The c-mycER^TAM ^technology acts at a protein rather than a transgene expression level. To mitigate the risk of the technology following intracerebral implantation of CTX0E03 cells it was important to investigate if c-mycER^TAM ^transcription level was a predictive measure of the recombinant protein level. Western blot analysis of the *in vivo *samples proved impossible because the c-MycER^TAM ^protein would be too dilute to detect in whole brain extracts. In addition, IHC analysis of the *in vivo *samples was impractical because of the substantial number of sections required to perform a global analysis of c-MycER^TAM ^protein expression. To overcome these challenges we developed and *in vitro *model of c-mycER^TAM ^silencing in CTX0E03 cells. The kinetics of transgene silencing *in vitro *was slower than that observed *in vivo*, which provided the ideal cell culture model to directly compare the progressive decline in c-mycER^TAM ^transcription with the translated protein level. Although we observed a decrease in the number of c-mycER^TAM ^positive cells by ICC, the relative percentage of positive cells did not closely follow relative transgene expression levels. These ICC findings further highlight the drawback of histological approaches because the cells were scored positive independent of the quantity of c-MycER^TAM ^protein present within the cell. On the other hand, when total c-MycER^TAM ^protein levels were measured by western blot they were found to decrease in parallel with transgene transcript level over the 1- to 4-week period. This data indicates that the c-mycER^TAM ^transcript and protein levels are closely linked in CTX0E03 cells.

## Conclusion

In conclusion, we have demonstrated that c-mycER^TAM ^transgene expression levels in CTX0E03 cells were significantly reduced *in vitro*, upon growth factor and 4-OHT withdrawal, and *in vivo *following implantation. We demonstrated that *in vivo *transgene silencing occurred through DNA methylation. In addition, the *in vitro *results showed that c-mycER^TAM ^transcription level was representative of the c-MycER^TAM ^protein level. The data demonstrate that uncontrolled *in vivo *cell growth arising from c-mycER^TAM ^technology is unlikely based on both the silencing of the c-mycER^TAM ^gene expression reported here and the specific requirement for the 4-OHT ligand to activate the c-MycER^TAM ^protein. Furthermore, these data demonstrate that the retroviral insertion of stable transgene constructs into human neural stem cells with the CMV promoter element is not an inherently unsafe procedure for the production of cellular therapies.

## Methods

### Ethics statement

Fetal tissue used to derive CTX0E03 cells was obtained in accordance with nationally (UK and/or USA) approved ethical and legal guidelines. All animal work was performed in accordance with the UK Animals (Scientific Procedures) Act (1986) and approved by the local animal Ethical Review Committee.

### Derivation of CTX0E03

The CTX0E03 clonal cell line was derived from human fetal brain cortical tissue of 12 weeks' gestation, as described previously [19]. Briefly, a mixed culture was initiated from the dissociated cortex tissue and was infected with an amphotropic replication-incompetent retroviral vector (pLNCX-2; Clontech) encoding the *c*-mycER^TAM ^transgene. The CTX0E03 clonal cell line was isolated using a ring cloning method. The CTX0E03 cells were routinely cultured as a monolayer on mouse laminin (Trevigen, AMS Biotechnology, UK) freshly coated flasks in serum free medium (RMM) containing the growth factor supplements 10 ng/ml bFGF (Peprotech), 20 ng/ml EGF (Peprotech), and 100 nM 4-OHT (Sigma). All experiments were carried out using CTX0E03 cells between passage number 33 to 37, which corresponds to a population doubling level (PDL) ranging between 65 to 75.

### MCAo and cell grafting

Male Sprague-Dawley rats (Charles River) were group housed with food and water ad libitum. Briefly, animals were subjected to 60 minutes of unilateral MCAo initiated under halothane anesthesia [29]. At 50 minutes into the occlusion, animals were evaluated for behavioral dysfunction (forelimb flexion and contralateral circling behavior). Animals that did not demonstrate dysfunction were removed from the study.

CTX0E03 cells were harvested and suspended at a concentration of ~50,000 cells/μl in HBSS-NAC (HBSS without calcium, magnesium and containing 0.5 mM N-acetyl cysteine; Sigma) and implanted 4-weeks ± 3 days after the occlusion. Each grafted animal received two 4.5 μl bolus injections (approximately 225,000 cells/site with control lesioned animals receiving vehicle only) over 68 sec with 4 min rest before withdrawal. Coordinates from bregma were as follows: Site 1) AP 0.0 mm, L -3.6 mm, V -5.5 mm (from skull surface at bregma); and Site 2) AP 0.0 mm, L +3.6 mm, V -5.5 mm (from skull surface at bregma). Animal brains were collected at 1-week (6 grafted + 2 non-grafted controls) or 4-weeks (6 grafted + 3 non-grafted controls) post-implantation. All animals were administered Cyclosporin A (20 mg/kg, Sandoz Pharmaceuticals) in cremaphore L (Sigma) a day prior to grafting, the day of grafting and then three times weekly for the remaining duration of the study. In addition, Methylprednisolone (Pharmacia Upjohn) was administered at 20 mg/kg for the first 14 days, 10 mg/kg for days 15–17 and 5 mg/kg for days 18–19 post-grafting.

### Alu sequence assay to detect and quantify CTX0E03 cells

The qPCR Alu assay was carried out using the LightCycler 480 and the Probes Master mix (Roche). Briefly, an average of 250 ng of genomic DNA was amplified using specific primers (Forward-TGAGGCAGGCGAATCGCTTGAA, Reverse-GACGGAGTTTCGCTCTTGTTG) and a FAM-labeled fluorogenic probe (CGCGATCTCGGCTCACTGCAACCTCCATCG) (PrimerDesign) against a conserved region of the human Alu-Sq sequence (Accession number U14573). The PCR was preformed under the following conditions: 95°C for 30 seconds, followed by 45 cycles of 95°C for 15 s, 58°C for 30 s, 72°C for 15 s. Quantification of the human CTX0E03 DNA in rat tissue was based on a standard curve using serial dilutions that ranged from 1 to 10000 CTX0E03 cells, calculated on the assumption that one human cell contains 6.6 pg of gDNA [30]. The standard curve was performed in the presence of 250 ng of rat gDNA.

### CTX0E03 c-mycER^TAM ^transgene transcription assay

Primers were designed to specifically detect the relative *c*-mycER^TAM ^transgene transcription level in CTX0E03 cells using the Roche Diagnostics Lightcycler LC480 system. Using SYBR Green Master mix (Roche) and primers (Forward-AAAGGCCCCCAAGGTAGTTA, Reverse- AAGGACAAGGCAGGGCTATT), which amplified the c-myc and estrogen receptor (ER) junction, qPCR was performed under the following conditions: 95°C for 5 s, followed by 45 cycles at 95°C for 10 s, 60°C for 20 s,72°C for 20 s, 82°C for 5 s, measuring the fluorescence. A melt curve analysis was also performed after the assay to check the specificity of the reaction using the following conditions: 95°C for 5 s, 65°C for 60 s (1°C increments) followed by 97°C continuous. In order to carry out absolute quantification of c-mycER^TAM ^transcription, a known concentration standard was required to plot a standard curve. Based on the assumption that CTX0E03 cell contains one genomic copy of the *c*-mycER^TAM ^transgene [19] serial dilutions ranging from 10 to 10,000 copies of c-mycER^TAM ^were used per reaction to produce the standard curve. The standard curve was performed in the presence of rat cDNA.

### Calculation of c-mycER^TAM ^transcription level *in vivo*

At 1- or 4-weeks post-implantation, the rat brains were removed, cut into 50–250 mg sections and snap frozen in liquid nitrogen. The gDNA and total RNA was then sequentially isolated from each tissue piece using the AllPrep DNA/RNA mini kit (Qiagen). Four tissue sections were processed per rat brain (i.e. two tissue sections encompassing each injection tract). In brief, the tissue samples were homogenised in the supplied lysis buffer according to sample weight. The lysed sample was processed first through an AllPrep DNA spin column, to isolate the gDNA, and subsequently through an RNeasy spin column to selectively isolate RNA. The gDNA and total RNA samples (1 μl/reaction) were then analyzed by qPCR for Alu signal and qRT-PCR for *c*-mycER^TAM^, respectively. Based on the Alu signal, the number of CTX0E03 cells present per reaction was calculated from the standard curve. Similarly, the number of *c*-mycER^TAM ^transcripts present per reaction was interpolated from the c-mycER^TAM ^copy number standard curve. A calculation was then carried out to correct for the amount analyzed and the total volume of tissue sample lysate (for example, 1 μl from a total of 650 μl). The corrected figures were then expressed as the number of *c*-mycER^TAM ^transcripts present over the number of CTX0E03 cells present in each tissue section. Standard error of the mean was calculated for all tissue sections found to contain cells (Microsoft Excel version 2003).

### Transgene methylation analysis by bisulfite sequencing

Bisulfite treatment of DNA followed by sequencing was used to determine the endogenous transgene promoter pattern of methylation in CTX0E03 cells. Methylation is implicated in repression of genetic activity and involves the addition of a methyl group to the carbon-5 position of cytosine residues of the dinucleotide CpG. Bisulfite sequencing is a technique used to identify specific gDNA methylation sites based on the fact that treatment of DNA with bisulfite converts cytosine residues to thymidine, but leaves 5-methylcytosine residues unaffected. Bisulfite treatment thus introduces a specific change in the DNA sequence which is dependent on the methylation status of individual cytosine residues. A bisulfite reaction was performed using Epitect Bisulfite (Qiagen). Up to 2 μg of genomic DNA was used for conversion with the bisulfite reagent. Bisulfite-converted DNA was used as a template for PCR amplification. PCR primers were designed using the web program METHPRIMER ([31]; ). Primers used were: Forward – GGGAGTTTGTTTTGGTATTAAAATTAA and Reverse – ACTACTACTAATAAAAATTCTCCTCCTC. PCR conditions were 94°C for 5 minutes, 30 cycles of 94°C for 30 seconds, 60°C for 1 min, and 72°C for 30 seconds followed by 10 min at 72°C. PCR fragments were cloned into TA-cloning vector using TOPO TA Cloning Kit for Sequencing (Invitrogen). Ten individual clones were sequenced by GATC Biotech (Germany) using the T3 universal PCR primers. The resulting sequences were analyzed using blast software . Sequence analysis that identified an unchanged cytosine base (i.e. protected from the bisulfite conversion reaction) was considered to be methylated, but a cytosine conversion to thymidine in the sequence indicated no methylation.

### *In vitro *characterisation: western blot and immunocytochemical analysis

*In vitro*, CTX0E03 cell samples were prepared by maintaining the cells in non-growth permitting medium alone, without the addition of EGF, bFGF, and 4-OHT, for 1- and 4-weeks. Western blot analysis for c-mycER^TAM ^protein in CTX0E03 cells was carried out using the whole-cell lysate. To prepare cell lysate, CTX0E03 cell monolayers in a T75 flask were rinsed with cold PBS (4°C), lysed with 750 μl 1× SDS Sample Buffer and dithiothreitol (DTT) Reducing Agent (PAGEgel Inc, AMS Biotechnology, UK), and collected in a 1.5 ml centrifuge tube. Samples were triturated multiple times to lyse and shear the sample, heated at 100°C for 2 min and centrifuged. Cell lysates were loaded onto a 4%–20% Tris-Tricine gel (PAGEgel Inc, AMS Biotechnology, UK), 10 μl per well. After electrophoresis, the protein was transferred onto a nitrocellulose membrane with 0.2 μm pore size by electroelution. Immunodetection was performed with a rabbit anti-ERα polyclonal or a mouse anti-c-Myc monoclonal antibody (Santa Cruz Biotechnology Inc, both used at 1:100). Transgene product was detected using horseradish peroxidase-conjugated anti rabbit-IgG (Cell Signaling Technology, 1:2000) or anti mouse-IgG (Pierce Biotechnology Inc., 1:800) secondary antibodies, as appropriate. The nitrocellulose membrane was then processed using chemiluminescence detection reagents (Thermo scientific). The blots were then stripped and reprobed using anti-α-tubulin (Sigma, 1:1000) to act as an internal loading level standard. Western blot images were captured using BioRad FluorS Imaging Unit and densitometry carried out using Scion Image software (Scion Corporation, version Alpha 4.0.3.2, USA).

Immunocytochemistry was carried out on CTX0E03 cells fixed in a 96-well plate (BD) using 4% PFA/4% sucrose in PBS for 15 min after 3 days growth (control) and following 1- and 4-weeks in medium containing no growth factors (EGF, bFGF) or 4-OHT. Anti-c-myc (1:100) and anti-ERα (1:500) were incubated in 1xPBS containing 1% normal goat serum (Vector laboratories) overnight at room temperature. After rinsing, the CTX0E03 cells were incubated for 1.5 h at room temperature using anti-mouse conjugated Alexa Fluor 488 (Invitrogen, 1:2000) for c-myc and anti-rabbit conjugated Alexa Fluor 568 (Invitrogen, 1:2500) for ERα. CTX0E03 cells were then counterstained by incubating with Hoechst 33342 (Sigma, 1 μM) for 2 min. Quantification of c-myc and ERα was carried out by manual counting of three representative fields using an Olympus IX70 fluorescent microscope.

## Authors' contributions

EM, LS, AH and JS conceived the experiments. LS, EM, RC, JH and PS designed and performed the experiments and analyzed the data. All authors contributed to the preparation of the written manuscript.

## References

[B1] Wong AM, Hodges H, Horsburgh K (2005). Neural stem cell grafts reduce the extent of neuronal damage in a mouse model of global ischaemia. Brain Res.

[B2] Hodges H, Nelson A, Virley D, Kershaw TR, Sinden JD (1997). Cognitive deficits induced by global cerebral ischaemia: prospects for transplant therapy. Pharmacol Biochem Behav.

[B3] Sinden JD, Rashid-Doubell F, Kershaw TR, Nelson A, Chadwick A, Jat PS, Noble MD, Hodges H, Gray JA (1997). Recovery of spatial learning by grafts of a conditionally immortalized hippocampal neuroepithelial cell line into the ischaemia-lesioned hippocampus. Neuroscience.

[B4] Modo M, Stroemer RP, Tang E, Patel S, Hodges H (2002). Effects of implantation site of stem cell grafts on behavioral recovery from stroke damage. Stroke.

[B5] Veizovic T, Beech JS, Stroemer RP, Watson WP, Hodges H (2001). Resolution of stroke deficits following contralateral grafts of conditionally immortal neuroepithelial stem cells. Stroke.

[B6] De Filippis L, Ferrari D, Rota Nodari L, Amati B, Snyder E, Vescovi AL (2008). Immortalization of human neural stem cells with the c-myc mutant T58A. PLoS ONE.

[B7] Bai Y, Hu Q, Li X, Wang Y, Lin C, Shen L, Li L (2004). Telomerase immortalization of human neural progenitor cells. Neuroreport.

[B8] Villa A, Navarro-Galve B, Bueno C, Franco S, Blasco MA, Martinez-Serrano A (2004). Long-term molecular and cellular stability of human neural stem cell lines. Exp Cell Res.

[B9] Donato R, Miljan EA, Hines SJ, Aouabdi S, Pollock K, Patel S, Edwards FA, Sinden JD (2007). Differential development of neuronal physiological responsiveness in two human neural stem cell lines. BMC Neurosci.

[B10] De Filippis L, Lamorte G, Snyder EY, Malgaroli A, Vescovi AL (2007). A novel, immortal, and multipotent human neural stem cell line generating functional neurons and oligodendrocytes. Stem Cells.

[B11] Cho T, Bae JH, Choi HB, Kim SS, McLarnon JG, Suh-Kim H, Kim SU, Min CK (2002). Human neural stem cells: electrophysiological properties of voltage-gated ion channels. Neuroreport.

[B12] Ryder EF, Snyder EY, Cepko CL (1990). Establishment and characterization of multipotent neural cell lines using retrovirus vector-mediated oncogene transfer. J Neurobiol.

[B13] Snyder EY, Deitcher DL, Walsh C, Arnold-Aldea S, Hartwieg EA, Cepko CL (1992). Multipotent neural cell lines can engraft and participate in development of mouse cerebellum. Cell.

[B14] Rao MS, Anderson DJ (1997). Immortalization and controlled in vitro differentiation of murine multipotent neural crest stem cells. J Neurobiol.

[B15] Jung M, Kramer EM, Muller T, Antonicek H, Trotter J (1998). Novel pluripotential neural progenitor lines exhibiting rapid controlled differentiation to neurotransmitter receptor-expressing neurons and glia. Eur J Neurosci.

[B16] Villa A, Snyder EY, Vescovi A, Martinez-Serrano A (2000). Establishment and properties of a growth factor-dependent, perpetual neural stem cell line from the human CNS. Exp Neurol.

[B17] Nakafuku M, Nakamura S (1995). Establishment and characterization of a multipotential neural cell line that can conditionally generate neurons, astrocytes, and oligodendrocytes in vitro. J Neurosci Res.

[B18] Miljan EA, Hines SJ, Pande P, Corteling RL, Hicks C, Zbarsky V, Umachandran M, Sowinski P, Richardson S, Tang E (2008). Implantation of c-mycERTAM immortalized human mesencephalic derived clonal cell lines ameliorates behaviour dysfunction in a rat model of Parkinson disease. Stem Cells Dev.

[B19] Pollock K, Stroemer P, Patel S, Stevanato L, Hope A, Miljan E, Dong Z, Hodges H, Price J, Sinden JD (2006). A conditionally immortal clonal stem cell line from human cortical neuroepithelium for the treatment of ischemic stroke. Exp Neurol.

[B20] Maurer J, Fuchs S, Jager R, Kurz B, Sommer L, Schorle H (2007). Establishment and controlled differentiation of neural crest stem cell lines using conditional transgenesis. Differentiation.

[B21] Littlewood TD, Hancock DC, Danielian PS, Parker MG, Evan GI (1995). A modified oestrogen receptor ligand-binding domain as an improved switch for the regulation of heterologous proteins. Nucleic Acids Res.

[B22] Stroemer P, Patel S, Hope A, Oliveira C, Pollock K, Sinden J The Neural Stem Cell Line CTX0E03 Promotes Behavioral Recovery and Endogenous Neurogenesis after Experimental Stroke in a Dose-Dependent Fashion. Neurorehab Neural Repair.

[B23] Zhang F, Thornhill SI, Howe SJ, Ulaganathan M, Schambach A, Sinclair J, Kinnon C, Gaspar HB, Antoniou M, Thrasher AJ (2007). Lentiviral vectors containing an enhancer-less ubiquitously acting chromatin opening element (UCOE) provide highly reproducible and stable transgene expression in hematopoietic cells. Blood.

[B24] Lee HJ, Kim KS, Kim EJ, Choi HB, Lee KH, Park IH, Ko Y, Jeong SW, Kim SU (2007). Brain transplantation of immortalized human neural stem cells promotes functional recovery in mouse intracerebral hemorrhage stroke model. Stem Cells.

[B25] Nicklas JA, Buel E (2003). Development of an Alu-based, real-time PCR method for quantitation of human DNA in forensic samples. J Forensic Sci.

[B26] Schneider T, Osl F, Friess T, Stockinger H, Scheuer WV (2002). Quantification of human Alu sequences by real-time PCR – an improved method to measure therapeutic efficacy of anti-metastatic drugs in human xenotransplants. Clin Exp Metastasis.

[B27] Lavie L, Kitova M, Maldener E, Meese E, Mayer J (2005). CpG methylation directly regulates transcriptional activity of the human endogenous retrovirus family HERV-K(HML-2). J Virol.

[B28] Strathdee G, Brown R (2002). Aberrant DNA methylation in cancer: potential clinical interventions. Expert Rev Mol Med.

[B29] Longa EZ, Weinstein PR, Carlson S, Cummins R (1989). Reversible middle cerebral artery occlusion without craniectomy in rats. Stroke.

[B30] Applied_Biosystems Creating standard curves with genomic DNA or plasmid DNA templates for use in quantitative PCR. http://www.appliedbiosystems.com/support/tutorials/pdf/quant_pcr.pdf.

[B31] Li LC, Dahiya R (2002). MethPrimer: designing primers for methylation PCRs. Bioinformatics.

[B32] Schroeder A, Mueller O, Stocker S, Salowsky R, Leiber M, Gassmann M, Lightfoot S, Menzel W, Granzow M, Ragg T (2006). The RIN: an RNA integrity number for assigning integrity values to RNA measurements. BMC Mol Biol.

